# Iron Oxide Colloidal Nanoclusters as Theranostic Vehicles and Their Interactions at the Cellular Level

**DOI:** 10.3390/nano8050315

**Published:** 2018-05-09

**Authors:** Athanasia Kostopoulou, Konstantinos Brintakis, Eirini Fragogeorgi, Amalia Anthousi, Liberato Manna, Sylvie Begin-Colin, Claire Billotey, Anthi Ranella, George Loudos, Irene Athanassakis, Alexandros Lappas

**Affiliations:** 1 Institute of Electronic Structure and Laser, Foundation for the Research and Technology, Hellas, Vassilika Vouton, 711 10 Heraklion, Greece; akosto@iesl.forth.gr (A.K.); kbrin@iesl.forth.gr (K.B.); ranthi@iesl.forth.gr (A.R.); 2 Institute of Nuclear & Radiological Sciences, Technology, Energy & Safety, NCSR “Demokritos”, 153 41 Aghia Paraskevi, Athens, Greece; fragkogeorgi@rrp.demokritos.gr; 3 Department of Biology, University of Crete, Vassilika Vouton, 710 03 Heraklion, Greece; Amalia.Anthousi@lstmed.ac.uk (A.A); athan@biology.uoc.gr (I.A.); 4 Istituto Italiano di Tecnologia, Via Morego 30, 16163 Genova, Italy; liberato.manna@iit.it; 5 Université de Strasbourg, CNRS, Institut de Physique et Chimie des Matériaux de Strasbourg, UMR 7504, F-67034 Strasbourg, France; sylvie.begin@ipcms.unistra.fr; 6 Université de Lyon, Université Jean Monnet, EA 3738, Ciblage Thérapeutique en Oncologie, UJM-UCBL-HCL, Hôpital E. Herriot, 5 place d’Arsonval, 69437 Lyon CEDEX 03, France; claire.billotey@univ-st-etienne.fr; 7 Bioemission Technology Solutions, Alexandras 116, 117 42 Athens, Greece; gloudos@teiath.gr; 8 Department of Biomedical Engineering, Technological Educational Institute, 122 10 Egaleo, Athens, Greece

**Keywords:** magnetic nanoclusters, multicore particle assembly, MRI contrast agents, scintigraphic imaging, nanoparticle biodistribution, cell interactions, immune system

## Abstract

Advances in surfactant-assisted chemical approaches have led the way for the exploitation of nanoscale inorganic particles in medical diagnosis and treatment. In this field, magnetically-driven multimodal nanotools that perform both detection and therapy, well-designed in size, shape and composition, are highly advantageous. Such a theranostic material—which entails the controlled assembly of smaller (maghemite) nanocrystals in a secondary motif that is highly dispersible in aqueous media—is discussed here. These surface functionalized, pomegranate-like ferrimagnetic nanoclusters (40–85 nm) are made of nanocrystal subunits that show a remarkable magnetic resonance imaging contrast efficiency, which is better than that of the superparamagnetic contrast agent Endorem^©^. Going beyond this attribute and with their demonstrated low cytotoxicity in hand, we examine the critical interaction of such nanoprobes with cells at different physiological environments. The time-dependent in vivo scintigraphic imaging of mice experimental models, combined with a biodistribution study, revealed the accumulation of nanoclusters in the spleen and liver. Moreover, the in vitro proliferation of spleen cells and cytokine production witnessed a size-selective regulation of immune system cells, inferring that smaller clusters induce mainly inflammatory activities, while larger ones induce anti-inflammatory actions. The preliminary findings corroborate that the modular chemistry of magnetic iron oxide nanoclusters stimulates unexplored pathways that could be driven to alter their function in favor of healthcare.

## 1. Introduction

Inorganic nanoparticulate systems, with optimized coating, have been utilized as effective multifunctional platforms in the complex environment encountered in biological and medical applications [1]. Moreover, multifunctional nanoarchitectures that are capable of magnetically-driven [2,3] diagnosis, drug delivery, and monitoring of therapeutic response are expected to play a significant role in the dawning era of personalized medicine; as such, much research effort has been devoted toward that goal [4]. For this purpose, the interactions between nanoparticles and cells are a key issue that has to be taken into account when the physicochemical features of such materials are optimized. Understanding the cellular processing of nanomaterials helps correlating their responsivity to physical stimuli and their effectiveness in biomedical applications [5]. The resulting system, when carefully synthesized with appropriate surface ligand groups, may gain additional targeted functionality to deliberately avoid, for example, the immune system recognition, and even inhibit or enhance the immune response [6].

In this rapidly changing research field, synthetically controlled magnetic nanosystems of different morphologies and chemical phases have been used for magnetic resonance-based diagnostics, with promising magnetic resonance imaging (MRI) capability [7,8,9,10]. Until now, the magnetic nanoparticles of 3*d* transition metal oxides, primarily of the ferro-spinel (cf. Fe_3-δ_Ο_4_, with end-members the magnetite and maghemite, at δ = 0 and δ ~ 1, respectively) type of nanostructures, which are capable of improving the negative (dark) contrast in *T*_2_-weighted images, have found their way into the clinical routine due to their biological compatibility [11]. However, size and phase polydispersity [12] due to underdeveloped synthesis protocols and the abundance of structural defects at thermodynamically unstable nanoscale interfaces [13] often impart limited function in such novel materials. As a result, it is likely that magnetic properties and contrast efficiencies could vary from one batch to another [14]. In the recent years though, rapid advances in wet chemical routes for the synthesis of size and shape-controlled surface-functionalized magnetic nanoplatforms [15] have led to better colloidal stability, improved crystallinity, and monodispersity, with consequent MR detection [16,17,18] beyond that of common superparamagnetic iron oxide (SPION; D_TEM_ ≤ 10 nm diameter) contrast agents [19].

In addition, magnetic nanoparticles can be good candidates as functional mediators for magnetic hyperthermia, addressing cancer treatment [20,21,22,23], or even as actuators for magnetically controlled drug release [24,25,26]. Moreover, nanomaterials as stimuli of the immune system provide an increasingly important, recent scientific area of interest. Novel strategies have been evaluated for immunosuppression, with nanoparticles playing a key role as delivery vehicles for small-molecule immunosuppressive compounds [27]. Furthermore, it has been suggested that they may act to trigger cancer immunotherapy, so that nanosystems activate the immune system to battle the tumor, instead of attacking the tumor themselves [28]. For these purposes, intense materials-design efforts based on nanochemistry have been implemented so that the subtle balance between imaging and therapeutic capabilities is achieved on the same nanoscale motif, which is commonly known as “theranostics” [29,30,31]. The multifunctional character of such a single nanoscale vector could open the field to alternative prognosis and the tailored cure of diseases, thus boosting personalized nanomedicine-based treatments.

Driven by the demand for optimized theranostic nanomaterials that may combine enhanced MRI contrast properties and superior heating efficiency, single and multi-step clustering [32] methods (e.g., silica-coated [33], liposome-encapsulated [34], polyethylene glycol (PEG) surface-coordinated [35] materials, etc.) have been exploited as alternative design avenues. To date, a steady improvement in wet chemistry protocols [36] permits the integration of the synthesis and assembly of magnetic nanocrystals, thus leading to colloidal cluster-like architectures (i.e., multicore particles with optimized dimensions and engineered magnetization) that are directly dispersible in aqueous media [37,38,39,40]. In this direction, it has been demonstrated that when iron oxide (maghemite, γ-Fe_2_O_3_) nanocrystals are grown and assembled in a polyacrylate-mediated environment [41,42,43], the nanoclusters’ magnetically-driven functionalities, such as magnetic resonance imaging [44] and magnetic hyperthermia [45], become practically useful for nanobiotechnology. For example, the relaxometric properties of these nanoprobes, when compared against the former [46] commercial SPION contrast agent Endorem^®^ [47], were found to reach a diagnostic quality that was four to five times better in *T*_2_-weighted MR imaging techniques. This attribute has been claimed to arise from their collective magnetic features, as they are tuned by the γ-Fe_2_O_3_ nanocrystals’ packing fraction within the colloidal nanoclusters (CNCs) themselves [43]. The latter is a characteristic that also advocates to the CNCs’ enhanced size-dependent hyperthermic response [45]. Effectively, our ability to drive the inorganic nanoparticles’ organization over secondary colloidal nanostructures (e.g., 40–100 nm) lead to new or enhanced properties due to the emergence of magnetic coupling mechanisms across their strongly correlated, individual building blocks [3,43].

Motivated by the potential of such iron oxide nanoclusters as a *T*_2_–type MRI contrast agent, here we bring a critical, early-stage view of the interactions between these vectors and cells. Such an insight would be a valuable asset, as cellular activity could also mediate the degree of theirresponsivity to physical stimuli. Nanoclusters with 40 nm to 85 nm diameters were chosen in order to study their size-dependent interactions under selected physiological conditions. For this, in vivo spatiotemporal nanocluster distribution has been investigated by scintigraphic imaging of experimental mice models upon their injection with radiolabeled CNCs. The biodistribution, binding, uptake, and degradation of the CNCs by healthy spleen cells were all examined through in vitro experiments. The nanocluster internalization in white blood cells has been demonstrated. Finally, the CNCs were tested for their direct and indirect impact on the immune system. Some efficacy against infections may be recognized, but further laboratory tests are necessary in order to uncover how these iron oxide nanoprobes may operate in a targeted fashion within the increasingly complex physiological environment of common health disorders. 

## 2. Materials and Methods

### 2.1. Biodistribution Experiments

The in vivo biodistribution study and Scintigraphic Imaging were performed at the NCSR Demokritos (Aghia-Paraskevi, Attica Prefecture, Greece), using female normal Swiss-Webster Albino mice (15–25 g) purchased from the Breeding Facilities of the NCSR Demokritos (Permit Number: EL 25 BIO 019, EL 25 BIO 020, EL 25 BIO 039). The protocol and all of the animal procedures were approved by the General Directorate of Veterinary Services (Athens, Attica Prefecture, Greece) and by the Bioethical Committee of the Institution (Permit number: EL 25 BIO 022) on the basis of the European Directive 2010/63/EU on the protection of animals used for experimental purposes.

For the radiolabeling and the biodistribution studies, maghemite CNCs of 73-nm diameter have been utilized.

#### 2.1.1. Radiolabeling

For the nanocluster radiolabeling, the short-lived and single photon γ-emitting metastable isotope of technetium, Tc (^99m^Tc), was used. Briefly, 100 μL of a fresh [^99m^Tc]NaΤcO_4_ generator (DrytecTM, GE Healthcare (US)) eluate (~1–2 mCi) was reduced by adding to it a volume of 40 μL of SnCl_2_ solution (1 mg/mL in 0.5 N HCl). The pH of the mixture was immediately adjusted to ~7 using a sodium hydrogen carbonate solution, NaHCO_3_ (0.5 M). Aliquots of the nanoclusters suspension (4 mg/mL) were added, and the mixture was then allowed to react at room temperature under gently stirring for 30 min. Radioactivity was measured by an Atomlab 100 Dose Calibrator (Biodex Medical Systems Inc., Shirley, NY, USA), and quality control of the [^99m^Tc]Tc-labelled nanoclusters was performed with silica gel chromatography paper iTLC-SG (Agilent Technologies, Santa Clara, CA, USA) using acetone and a mixture of pyridine:acetic acid:water (3:5:1.5) as the mobile phases. The strips were left to dry, and then were cut into two or three pieces, and the radioactivity content of each piece was measured in a gamma counter. With the combination of these two systems, the percentage of the produced [^99m^Tc]Tc-CNC was calculated. The chemicals and reagents used were of analytical grade [48].

#### 2.1.2. Stability Study

A stability assay was performed at 0 min, 1 min, 3 min, and 24 h post-incubation of [^99m^Tc]Tc-CNCs with an excess amount of the competitive ligand of cysteine at two different concentrations (1 mM and 100 mM) for ^99m^Tc, and in an aqueous solution (saline (0.9% *v*/*v*)). The time-dependent increase of any free radioisotope was determined by using saline as the mobile phase system in ITLC-SG.

#### 2.1.3. Animal Studies

*(a)*  *Biodistribution*

The ex-vivo profile in normal mice was studied by intravenous injections with 100 μL, ~0.37 MBq (~0.1 mCi) of the radiolabeled CNCs in saline (pH: 7.0), via the tail vein. Following that, animals were sacrificed at pre-determined time intervals of 5 min, 60 min, and 24 h post-injection (p.i.), and the main organs or tissues were removed, weighed, and counted together with blood samples, muscle and urine, by a γ-counter system (Cobra II, from Canberra Packard Inc, Rockford, IL, USA). In comparison to a standard of the injected solution, results were expressed as a percentage of the injected dose (%ID) per gram of each organ or tissue. For total blood radioactivity calculations, blood is assumed to be 7% of the total body weight.

*(b)*  *Scintigraphic imaging*

The imaging studies were performed on a dedicated benchtop mouse-sized gamma camera (γ-eye by BET Solutions) [49], in combination with the X-ray part of a custom-made trimodal system [50]. The gamma camera is based on two position-sensitive photomultiplier tubes, coupled to a CsI (Na) pixelated scintillator and a low-energy lead collimator with parallel hexagonal holes. The system’s field of view is 5 × 10 cm^2^, its spatial resolution is 1.7 mm, and its energy resolution is ~19%. The X-ray system consists of an X-ray tube and a complementary metal-oxide-semiconductor (CMOS) flat panel sensor, C10900D, (Hamamatsu Photonics K.K., Hamamatsu, Japan), separated by a distance of 30 cm. The minimum pixel size is equal to 0.1 mm, and the active area is approximately 12 × 12 cm^2^.

For the scintigraphy imaging studies, 100 μL, ~0.37 MBq (~0.1 mCi) corresponding to 5 μg of radiolabeled CNCs were administered through the tail vein prior to anesthesia in normal mice. Anesthetization was performed intraperitoneally (i.p.) with 100 μL/10 g body weight of a stock solution containing 10% ketamine–hydrochloride (100 mg/mL) and 5% xylazine–hydrochloride (20 mg/mL) prior to scanning. Then, mice were positioned on the animal bed at a <0.5 cm distance from the camera head to allow whole body imaging with maximum spatial resolution. Successive 2-min frames were collected for up to 1 h p.i. for the intravenously injected mice, and after the first hour, static images were also acquired at 3 h and 24 h p.i. Regions of interest (ROIs) were drawn on major organs of interest; then, these ROIs were applied to the individual frames to provide semi-quantitative time activity curves. The ratio of counts in a ROI over the total number of counts is proportional to the radioactivity percentage per organ.

Upon completion of the scintigraphy imaging, X-ray images were also acquired at the exact same mouse positioning to act as an anatomical guide for the organs’ exact locations. The X-ray imaging parameters were set to 35 kVp, 500 μA, and 0.1 s exposure time. Fusion between scintigraphy and X-ray images was performed semi-automatically through an in-house standard procedure.

### 2.2. Materials and Synthesis of Nanoclusters

All of the reagents were used as received without further purification. Anhydrous iron chloride (FeCl_3_, 98%) was purchased from Alfa Aesar (Karlsruhe, Germany). Anhydrous sodium hydroxide (NaOH, 98%) and polyacrylic acid (PAA, Mw = 1800) were purchased from Sigma Aldrich (St. Louis, MO, USA), while diethylene glycol (DEG, (HOCH_2_CH_2_)_2_O) of reagent (<99.7%) and laboratory (<99.5%) grades were purchased from Fisher Scientific (Waltham, MA USA). The absolute ethanol was purchased from Sigma Aldrich.

The colloidal maghemite CNCs were synthesized using a high-temperature, polyol-based chemical protocol with iron chloride as the metal precursor, sodium hydroxide as the reductive medium, and polyacrylic acid as the capping agent. Appropriate molar quantities of the reagents were reacted at 220 °C under Argon atmosphere in a three-neck flask that was equipped with immersion temperature probes and digitally-controlled heating mantles. The reactor was connected through a reflux condenser to a Schlenk line setup. All of the reactants (i.e., FeCl_3_, NaOH, PAA, DEG), except ethanol, were stored and handled in an Argon recirculating anaerobic glove box (MBRAUN, UNILab, Garching, Germany). Details of the synthetic procedure and the route followed to optimize the nanoclusters’ size are published elsewhere [43,44].

### 2.3. In Vitro Experiments

#### 2.3.1. Animal Models

BALB/c (H-2^d^) inbred mice [51] were purchased from Charles River (Milan, Italy) and bred in the animal facility of the Department of Biology at the University of Crete (Crete, Greece, EL91-BIObr-09) under standard conditions of temperature (18–25 °C), humidity (45–50%), and photoperiod of 12 h light and 12 h dark. Males and females 4–8 weeks of age were handled according to the international and national bioethical rules and conformed to the bioethics regulations following the EU Directive 2010/63/EU for animal experiments. Mice were sacrificed by cervical dislocation, and spleens were removed under antiseptic conditions.

#### 2.3.2. Cell Cultures

*(a)*  *Spleen cells*

Spleens were dissected from the abdominal cavity of the BALB/c mice and put in single cell suspension in Hanks’ Balanced Salt Solution (HBSS) (Biosera, Kansas City, KS, USA). The isolated spleen cells were resuspended in Dulbecco’s Modified Eagle’s Medium (DMEM) (Biosera, Nuaille, France) supplemented with 10% fetal bovine serum (FBS, Gibco, Grand Island, NY, USA), and cultured in the appropriate culture plates (Sarstedt, Numbrecht, Germany) at the concentration of 1 × 10^6^ cells/mL, with or without CNCs. Two sizes of CNCs have been used: a small (40 nm) and a large (85 nm) one, in three different concentrations (100 μg Fe/mL, 200 μg Fe/mL, and 400 μg Fe/mL).

*(b)*  *Macrophage and lymphocyte isolation*

Spleen cells were isolated as mentioned above, resuspended in DMEM medium supplemented with 10% FBS, and cultivated in 10-mm Petri dishes (Sarstedt) overnight (16–18 h) at 37 °C in CO_2_ atmosphere. At the end of the incubation period, the culture supernatants, containing the red and white spleen cells, were transferred into new plates at the concentration of 1 × 10^6^ cells/mL in DMEM–10% FBS culture medium, with or without CNCs. The remaining adherent macrophages were scraped off the plate, washed, and cultured in fresh DMEM–10% FBS medium, with or without CNCs. Two sizes of nanocarriers have been used, a small (40 nm) and a large (85 nm) one, in three different concentrations (100 μg Fe/mL, 200 μg Fe/mL, and 400 μg Fe/mL).

*(c)*  *Internalization of nanoclusters*

CNCs were tested for their ability to accumulate into red and white cells and potentially lead to cell apoptosis or necrosis. Spleen cells were incubated for 15 min, 30 min, 60 min, and 90 min with three different CNCs concentrations at 37 °C in an atmosphere of 5% CO_2_. Binding was evaluated by submitting cell suspension to magnet isolation, eliminating non-bound cells, washing, and subsequent cell counting.

*(d)*  *Cytokine production*

Indirect ELISA was performed in order to verify the ability of nanoclusters to induce the cytokine production by spleen cells. Specifically, spleen cells were isolated as mentioned above, cultured with or without CNCs for 48 h, and their culture supernatants (1:2 dilutions) were examined for the presence of IL-2, IL-4, IL-10, TNF-α, and IFN-γ.

Briefly, samples were diluted in 0.05 Μ of NaHCO_3_ and 0.05 M of Na_2_CO_3_ in H_2_O dist (pH: 9.6; coating buffer), coated in 96-well flat bottom plates (Sarstedt), incubated overnight at 4 °C, and washed three times in 5% Tween-20. The remaining protein-free sites in the plate were blocked by 200 μL/well of blocking solution, upon incubation for 2 h at room temperature. The blocking solution consists of 2% w/v Bovine Serum Albumin (BSA, Fraction V, Applichem, Saxony-Anhalt, Germany) in Phosphate-Buffered Saline (PBS, Gibco, Grand Island, NY, USA). After washing three times, 100 μL of test antibodies, including anti-IL-2, anti-IL-4, anti-IL-10, anti-TNFα, or anti-IFN-γ (0.1 μg/mL; Immunotools, Friesoythe, Germany) were diluted in 0.1% PBS-BSA and incubated for 2 h at room temperature. Extensive washing of the plate was followed by the addition of 100 μL of goat anti-mouse IgG coupled to horseradish peroxidase and incubation for 1 h at room temperature, in dark. Finally, the reaction was developed by adding 100 μL/well of tetramethylbenzidine-H_2_O_2_ (TMB Substrate Set, TMB Substrate A and B, Biolegent, San Diego, CA, USA) for 5 min. The enzymatic reaction was stopped with 50 mL of H_2_SO_4_ (1 M). Optical density (OD) was measured at 450 nm using a Titertec ELISA photometer (Digiscan, ASYSHitech, GmbH; Engendorf, Austria).

*(e)*  *Cell proliferation assay*

Bulk spleen cells, macrophages, or lymphocytes were cultured in 96-well V-bottomed plates (Sarstedt, Numbrecht, Germany) at the concentration of 1 × 10^6^ cells/well in DMEM culture medium (Biosera, Kansas City, MO, USA) supplemented with 10% fetal bovine serum (FBS, Gibco) at a final volume of 200 μL with or without the two different sizes of CNCs (in three different concentrations), and processed for tritiated thymidine (^3^HTdR) incorporation assays after three days of culture. The cultures were pulsed with 1 μCi of ^3^HTdR (ICN, Costa Mesa, CA, USA) 18 h prior to harvest. After transferring the cells onto cellulose filters, these were put in scintillation fluid (toluene-omnifluor; 1.38 g/L, NEN), and counted using a beta-counter (LKB-Wallac, Turku, Finland).

*(f) Statistical analysis*

Data were analyzed with two-tailed paired (in vitro experiments) or unpaired (in vivo experiments) student’s *t*-tests. *p*-values < 0.05 were considered significant (*), values < 0.01 were considered very significant (**), and values < 0.001 and < 0.0001 were considered highly significant (*** and ****). Statistics were performed using GraphPad Prism 6.01 (Graphpad Software, La Jolla, CA, USA).

### 2.4. Characterization Methods

#### 2.4.1. TEM Imaging

*(a)*  *TEM imaging of the inorganic nanomaterials*

Low magnification images were recorded on a LaB_6_ JEOL 2100 electron microscope (JEOL Ltd., Tokyo, Japan) operating at an accelerating voltage of 200 kV. For the purposes of the TEM analysis, a drop of a diluted colloidal nanocluster aqueous solution was deposited onto a carbon-coated copper TEM grid, and then, the water was allowed to evaporate. All of the images were recorded by the Gatan ORIUSTM SC 1000 CCD camera. In order to estimate the average diameter (D_TEM_), statistical analysis was carried out on several low magnification TEM images, with the help of the dedicated software ImageJ (version 1.52a) [52].

*(b)*  *TEM imaging of the biological samples*

Cells were fixed in 2% glutaraldehyde, 2% paraformaldehyde for 24 h at 4 °C, washed in 0.1 M of sodium cacodylate buffer (PH: 7.4), post-fixed in 2% OsO_4_ in 0.1 M of sodium cacodylate buffer for 60 min at 4 °C, and dehydrated in increasing concentrations of alcohol. The samples were impregnated with propylene oxide and embedded in epoxy resin embedding media. Then, ultrathin sections were cut with an LKB ultratome V-2088 (Οlympus, Bromma, Sweden) and placed on a TEM grid that was post-stained with uranyl acetate and lead citrate. Images were obtained using on a LaB_6_ JEOL 2100 electron microscope operating at an accelerating voltage of 80 kV.

*(c)*  *SEM imaging and EDS analysis*

With the purpose of evaluating renal (urine) elimination of the inorganic nanoparticulate systems utilized in this study, we used a JEOL scanning electron microscope (SEM; model JSM 6390, Jeol USA Inc, Peabody, MA, USA) equipped with a tungsten filament operating at 20 kV. For the detection of iron-based moieties, 5 μL of a purified aqueous solution of nanoclusters was intravenously injected in a mouse model, and specimens of the excreted urine were carefully collected. The urine volume was left to dry on a glass slide under the influence of a small permanent magnet. The utility of the latter was crucial in order to increase the density of the magnetic nanomaterial and permit its spatial detection, as well as its elemental analysis by means of energy-dispersive X-ray spectroscopy (EDS).

#### 2.4.2. Z-Potential and Dynamic Light Scattering (DLS) Analysis

Such measurements were carried out using a Malvern Instruments Zeta-Sizer (Malvern Panalytical Ltd., Malvern, UK) equipped with a 4.0 mW He-Ne laser operating at 633 nm and an avalanche photodiode detector. When the nanoscale particle size is derived from DLS, the size distribution by “number” is presented for comparison against TEM-measured dimensions of inorganic moieties, while size distribution by “intensity” is used when the hydrodynamic diameter is of interest.

#### 2.4.3. Magnetic Measurements

The hysteresis loop, M(H), of the as-synthesized CNCs samples was recorded at room temperature by means of a Superconducting Quantum Interference Device (SQUID) magnetometer (Quantum Design MPMS XL5, San Diego, CA, USA). This magnetic measurement was performed by sweeping the field between −1 ≤ H ≤ +1 Tesla, and utilizing a powder sample that was produced after drying a small quantity of the purified colloidal dispersion of the nanoclusters.

#### 2.4.4. Relaxivity Measurements and *T*_1_ & *T*_2_ Weighted Phantoms

Iron oxide nanoparticles are commercially used as *T*_2_-type contrast agents (CAs) in magnetic resonance imaging (MRI). *T*_2_ CAs, which are commonly called negative CAs, reduce the transverse *T*_2_ relaxation time and diminish the signal intensity in *T*_2_-weighted images. The efficacy of a CA is evaluated in terms of the relaxivity (*r_i_*; i.e., a measure of the water-proton relaxation rate), but the ratio *r*_2_*/r*_1_ should also be considered in order to determine whether a material is a good *T*_1_ or *T*_2_ CA candidate. Therefore, *T*_2_ agents are characterized by high *r_2_* relaxivity and a high *r*_2_*/r*_1_ ratio.[53] For this purpose, *T*_1_ and *T*_2_ measurements were performed on a Bruker Minispectrometer mq20 (Karlsrushe, Germany), working at a Larmor frequency of 20 MHz (0.47 Tesla) at 37 °C. The longitudinal and transverse relaxation times, *T*_1_ and *T*_2_, respectively, were measured on three diluted suspensions. The mean relaxation times were obtained from three measurements on suspensions with a given Fe concentration, *C* (the initial purified solution, *C* ≅ 8 mM Fe, was defined by Inductively coupled plasma atomic emission spectroscopy (ICP-AES) from nanoclusters of D_TEM_ ~73 nm). The contribution of water is removed from the obtained mean values, and the quantities ${\left(\frac{1}{T_{i}}\right)}_{meas}-{\left(\frac{1}{T_{i}}\right)}_{water}$ are plotted as a function of the Fe concentration, leading to a linear relation. The relaxivities *r*_1_ and *r*_2_ are then determined through the slope of the concentration (*C*) dependence of *r*_1_ = 1/*T*_1_, *r*_2_ = 1/*T*_2_.

The capability of the iron oxide nanoclusters for MRI contrast effects was further evaluated in vitro by using a small animal dedicated 7 Tesla (300 MHz) MR Biospec™ system (Brüker, Wissenbourg, France) equipped with 400 mT/m gradient, while imaging 900 microliter water-based samples containing a progressively reduced iron concentration. This scanner system utilized an inversion recovery FLASH (IR-FLASH) imaging sequence with varying IR; this allowed evaluating the spin-lattice (*r*_1_) and spin-spin (*r*_2_) relaxivities, which are defined by the concentration-independent values of the relaxation rate enhancement for the longitudinal and transverse magnetization components, respectively. Then, common imaging sequences weighted with *T*_1w_ (spin-echo sequence: TR/TE = 500/12 msec) and *T*_2w_ (multi spin-echo sequence: TR/TE = 2000/70 msec) were acquired in order to assess the *T*_1_ and *T*_2_-mediated effects as a function of the Fe concentration. The value of the MR contrast enhancement (EHC), against a water reference sample, was calculated as: $\mathrm{EHC}\left(\%\right)w=\frac{\left(\mathrm{Signal}\;\mathrm{value}\;\mathrm{of}\;\mathrm{iron}\;\mathrm{oxide}\;\mathrm{nanoclusters}\right)-\left({\mathrm{Signal}\;\mathrm{value}\;\mathrm{of}\;H}_{2}O\right)}{{\mathrm{Signal}\;\mathrm{value}\;\mathrm{of}\;H}_{2}O}\text{×}100$; a positive value of the EHC indicates a *T*_1_ effect, while a negative EHC value indicates a *T*_2_ effect.

## 3. Results

### 3.1. Functional Colloidal Maghemite Nanoclusters and their MRI Detection Capability

A high-temperature polyol-based synthesis approach has been adapted for the nucleation and growth of size-tailored (40 nm, 73 nm, and 85 nm) colloidal nanoclusters of maghemite nanocrystals after careful modification of the water content during the chemical synthesis. This is a single-step approach where the subtle impact of water is justified if we consider the higher affinity of the water molecules to coordinate stronger the surface metal cations, as compared to the affinity of the carboxylate groups (–COO^–^) of the polyacrylic acid (PAA). The purposeful choice of PAA as a capping agent makes the cluster surface highly charged (vide infra), thus offering stable aqueous colloidal solutions for further exploration in biomedical applications. Additional details on the nanocluster synthesis and purification have been published in earlier reports [43].

**Figure 1:**
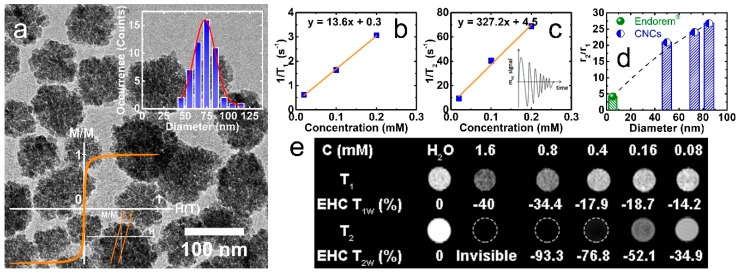
As-synthesized maghemite nanoclusters (D_TEM_ ~73 nm) in a low-magnification bright-field TEM image (**a**), the iron concentration (*C = x*) dependence of the proton NMR 1/*T*_1_ (**b**) and 1/*T*_2_ (**c**) at room temperature (H = 0.47 T), the enhancement of the relaxivity ratio, *r*_2_/*r*_1_, for variable diameter nanoclusters against Endorem^®^ (H = 0.47 T; the data for the 50 nm and 85 nm nanoclusters were reproduced with permission from [44]) (**d**), and (**e**) the *T*_1_ and *T*_2_-weighted MR ghost images (H = 7 T) and the enhancement contrast ratio (EHC) as a function of the aqueous solution Fe concentration, *C*. Insets: (**a**) the normalized hysteresis loop (M/M_S_) at 300 K (bottom left) and nanocluster Gaussian size distribution (upper right); (**c**) schematic of the efficient water-proton nuclear magnetic moment transverse relaxation (*T*_2_; dark contrast) in the vicinity of magnetic nanoprobes.

The nanoclusters are uniform in size/shape, and almost spherical. They have a pomegranate-like morphology, and no aggregation exists between the nanocluster units, while the colloidal solution shows no individual nanocrystals left out of them. A typical specimen, such as that used in the present work, is depicted in the low-magnification TEM image of Figure 1a (average diameter, D_TEM_= 72.9 ± 14.5 nm). A hysteresis loop recorded for the dried powder of CNCs at room temperature shows weak ferrimagnetic (FiM) behavior (small coercive field, H_C_ ~ 2 Oe) (Figure 1a, left inset). Earlier experimental and theoretical investigations have pointed out that the nature of magnetism and the increased saturation magnetization (*M*_S_ = 73 emu/g γ-Fe_2_O_3_) of such nanoassemblies are due to the synergistic character of the intracluster dipolar magnetic interactions [43].

In view of this, the clusters have the capability to become easily magnetized under a moderate external magnetic field, such as that utilized in MR scanners, thus allowing the nanoprobes to create a substantial perturbation of the local dipolar magnetic field in their vicinity. The purposeful clustering of small individual nanoparticles in secondary, well-controlled nanostructures raises *M*_S_. In turn, this leads to the enhancement of the water-proton nuclear magnetic moment transverse relaxation rate [3], as it is directly related to the saturation magnetization (*r*_2_ ~ *d*^2^ *M*_V_^2^/*φ*, where *d* is the inorganic moiety diameter, *M*_V_ = *M*_S_ *φ* is the overall magnetization, and *φ* is the intra-aggregate magnetic material volume fraction) [54]. With the intention to evaluate the nanocluster contrast capability, the slopes of 1/*T*_1_ and 1/*T*_2_ against the varying nanocluster concentration, *C*, were determined to estimate the longitudinal (*r*_1_) and transverse (*r*_2_) relaxation rates, namely as 13.6 mM^−1^ s^−1^ and 327 mM^−1^ s^−1^, respectively (Figure 1b,c). Interestingly, the relaxivity ratio *r*_2_/*r*_1_ for the 73-nm nanoclusters is about six times higher than that of the superparamagnetic contrast agent Endorem^®^ (*r*_2_/*r*_1_ = 93/22 ~ 4.2), and consistently enhanced against the related SPION samples of D_TEM_ ~5–12 nm diameter (*r*_2_/*r*_1_ ~ 1.6–5) [55]. Although the high *r*_2_ and *r*_2_/*r*_1_ may be considered as significant attributes for the CNCs, one must also take into account that the apparent improved contrast efficiency also arrives with likely drawbacks arising from their intrinsic contrast-generating mechanism. Namely, as the negative contrast in *T*_2_-weighted MR images involves a signal loss process (i.e., *T*_2_ contrast shows as a darkened area), this may prevent their use in low signal body regions, in organs with intrinsic high magnetic susceptibility (e.g., lung), or in the presence of hemorrhagic events [56]. To overcome such challenges, modifications of the diagnostic techniques (e.g., entailing off-resonance pulse sequences) have been proposed in order to generate a positive (bright) contrast and greater contrast-to-noise ratio in the presence of the strong magnetization carried by iron oxide nanoprobes [57].

The size-dependent evolution of the nanocluster relaxivity ratio at a low field (H = 0.47 T) is plotted in Figure 1d. The result provides experimental evidence that in this crystallographically-oriented assembly of nanocrystals (as defined by a characteristic spot pattern—similar to that of a single crystal specimen—attained by selected area electron diffraction of individual nanocluster entities) [43,44], the magnetic crystals’ spin moments are coupled to generate a strong (perturbing) local dipolar field around them, with consequent MR contrast benefits [3]. This might be expected in the motional averaging regime (Δ*ω τ*_D_ < 1, with Δ*ω* as the angular frequency shift, and τ_D_ as the ^1^H-translational diffusion time), where the water protons experience varying strength in the local magnetic fields. However, in the case of the static dephasing regime (Δ*ω τ*_D_ > 1), where these iron oxide CNCs belong [44], as the field fluctuations are negligible, it is the intra-aggregate magnetic material volume fraction (e.g., for related nanocluster specimens of D_TEM_ ~ 50 nm and 85 nm, *φ* = 0.60 and 0.72) that raises the magnetization (*M*v = *M*s *φ*) and boosts the *r*_2_ far and above that obtained with loosely-coupled (e.g., for Endorem^®^, *φ* = 0.23) or individual SPION samples (D_TEM_ ~ 5–12 nm) [54]. Beyond this regime, theory predicts and experiments have verified that transverse relaxivities tend to become suppressed [39,58].

The contrast enhancement capability of CNCs was further evaluated in vitro by MR ghost images (Figure 1e) taken at a 7 T scanner. The images indicated a very strong *T*_2_ effect, as illustrated by the intense negative *T*_2w_ contrast. The enhancement contrast ratio (EHC) measurements as a function of the iron concentration (Figure 1e) evidenced a very important *T*_2_ effect, even when the iron concentration was lowered significantly, namely: EHC *T*_2w_= −34.9% (−4.2%, at even stronger dilution) at 8 × 10^–2^ mM (4 × 10^−5^ mM). Furthermore, MR images with an iron concentration larger than 1.6 mM were not detectable due to the dramatic signal inhibition, preventing their EHC quantification (superior to 100%). The high negative *T*_2w_ EHC obtained for the CNCs at very low iron concentrations, despite the intense magnetic field (7 T) utilized, corroborate the high contrast power of these nanoclusters. As far as the *T*_1w_ images are concerned, no signal is observed at low concentrations, but at high concentrations, a hyposignal is noticed, with an EHC *T*_1w_ percentage of −40% related to the *T*_2_ effect at 1.6 mM of Fe concentration (Figure 1e). The intense *T*_2_ effect at high concentrations can be related to the strong *T*_2_ shortening, and also to the near proximity of the CNCs that is likely taking place at high concentrations [18,59].

### 3.2. Time-Dependent Nanocluster Biodistribution and Their Excretion through Urine

The promising magneto-structural features of the nanoclusters are of fundamental importance, but when new nanoparticulate colloids are to be utilized as MRI *T*_2_ contrast agents, additional insights are sought. For this, the in vivo spatiotemporal nanocluster distribution, as well as their excretion from an organism through the urine, would be of prime interest. Imaging studies for the nanocluster biodistribution were performed on a dedicated benchtop mouse-sized gamma camera (γ-eye by BET Solutions) [48], in combination with the X-ray part of a custom-made trimodal system [50]. A ^99m^Tc SPECT radionuclide was selected for binding due to its ideal physicochemical properties (i.e., half-life, photon energy), wide availability, and ability to give high-quality images. The greatest benefit of this technique is that through imaging, the pattern of the nanoclusters’ deposition to the rich macrophage organs (liver and spleen) was determined non-invasively by studying a series of time points in the same animal. Thus, an accurate and fast quantitative analysis of nanoclusters’ fate was conducted, while the number of animals that were used for dissection experiments was minimized. The fusion of functional scintigraphic images with the anatomical ones of the X-ray part provided more comprehensive information about CNCs’ in vivo profile.

**Figure 2:**
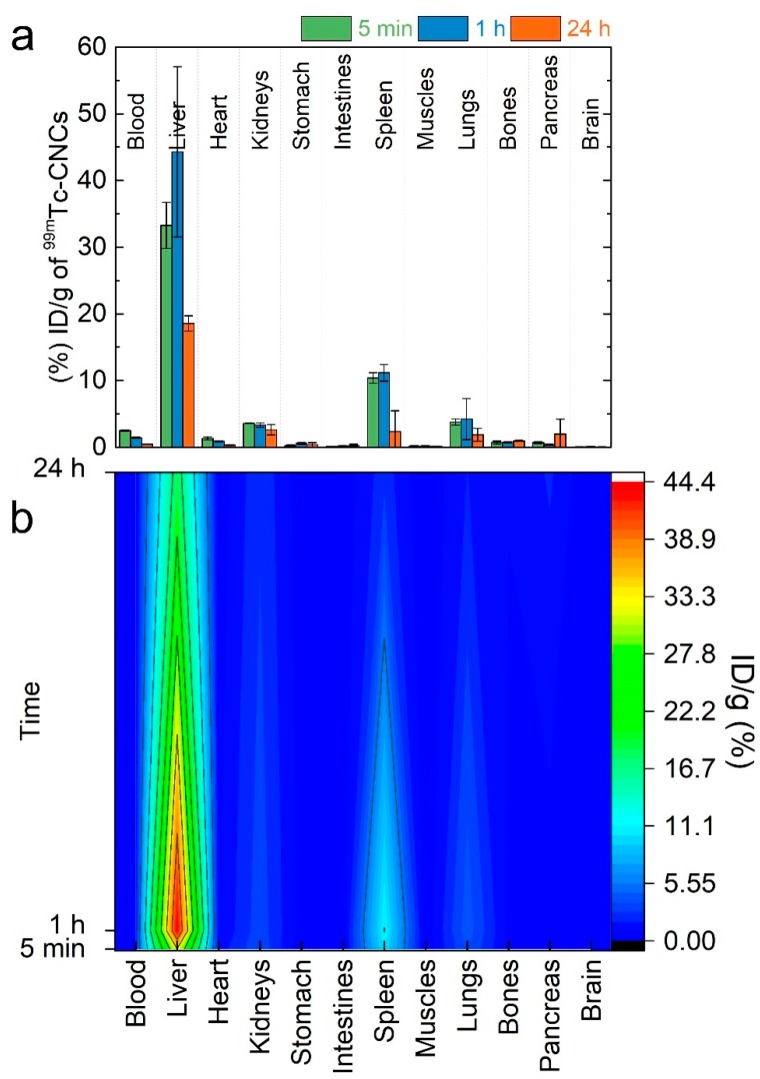
(**a**) Biodistribution data of Swiss-Webster mice intravenously injected with [^99m^Tc] Tc-colloidal nanoclusters (CNCs) (D_TEM_ ~73 nm). The values are expressed as a percentage (%) of injected dose (ID) per gram (g), at 5 min, 1 h, and 24 h post-injection (p.i.) (means ± standard deviation; (*n* = 2)); (**b**) Contour plot of ID/g (%) for the studied organs and the decrease of radioactivity over time.

The radiolabeling of CNCs (D_TEM_ ~ 73 nm) was performed by using a short-lived and single photon γ-emitting metastable isotope of technetium via a direct labeling approach [48]. The radiolabeled CNCs were intravenously injected to mice, and their biodistribution was studied at time intervals of 5 min, 1 h, and 24 h post-injection (p.i.) (Figure 2a). The highest blood values were observed at the earlier studied time-point of 5 min p.i., but even so, the percentage of CNCs was lower than 3%. High localization of the radiolabeled CNCs was seen in the tissues of the reticuloendothelial system (RES), such as the liver and spleen (Figure 2a). The associated higher radioactivity, and consequently the higher CNCs concentration in both organs, was observed in the time-interval of the first hour (Figure 2). Radioactivity was observed neither in the thyroid gland (image analysis) nor in the stomach (biodistribution study). This could be an indication of the stability of the radiolabeled CNCs, because it was previously found that free pertechnetate (or unreduced) [^99m^Tc]TcO_4_^-^ was mainly localized in these organs [60]. Beside this, the lung uptake was relatively low, indicating that the formation of aggregates in vivo, which could be irreversibly trapped in lung capillaries, was not significant. The detection of labeled CNCs was minimized to the liver and spleen after 24 h p.i., as the half-life of ^99m^Tc lays in 6 h (Figure 2b contour plot).

**Figure 3:**
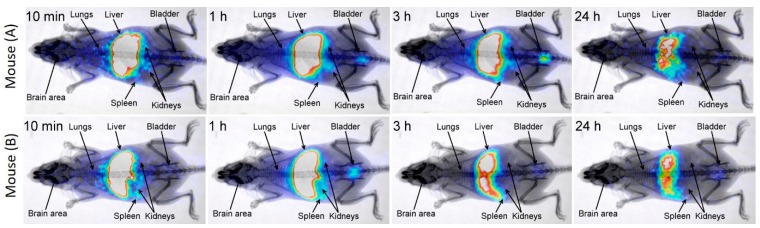
Static scintigraphy/X-ray images of two healthy Swiss-Webster Albino mice (**A** and **B**) intravenously injected (i.v.) via the tail vein with 5 μg of ^99m^Tc-labeled CNCs (~0.1 mCi) at 10 min, 1 h, 3 h, and 24 h post-injection (p.i.).

Scintigraphic dynamic imaging up to 1 h p.i. and static imaging at 3 h and 24 h p.i. confirmed the above ex vivo findings (Figure 3). However, radioactivity in organs that partially overlap (e.g., lungs/liver, liver/spleen) cannot be accurately depicted, and only indicative information is provided. It is worth noting that radioactivity was clearly detected in the bladder from 1 h p.i. This could be easily attributed to the detached ^99m^Tc from the nanoclusters, but this seems not to be the case. In line with this is the apparent firmness of the [^99m^Tc]Tc-CNCs conjugates, as is corroborated by their stability study. Radiolabeled CNCs showed increased stability (>90%) at room temperature, even after 3 h of saline solution preparation, while they remained intact (~65%) up to 24 h (Figure S1). Following that, the addition of an excess amount of the competitive ligand, cysteine, for ^99m^Tc at 1 mM, indicated that more than 70% of the labeled CNCs still remained intact. The specimen was shown to be 40 % intact from *t* = 0 s, even when a 100-fold higher concentration (100 mM) was used. In view of the [^99m^Tc]Tc-CNCs stability, and in order to verify the origins of the observed radioactivity in the bladder, careful ex-situ scanning electron microscopy, with energy-dispersive X-ray spectroscopic analysis (SEM-EDS) were also planned. For this, bare CNCs were intravenously injected in a mouse model, and the urine was collected and studied via optical as well as scanning electron microscopies (Figure S2a,b). The detection of iron-based species in the EDS maps inferred that the CNCs could be excreted through urine (Figure S2c,d). Overall, the stability study of the radiolabeled CNCs, together with an SEM-EDS analysis of collected urine, corroborate that the radioactivity signal from the bladder is likely due to conjugates of [^99m^Tc]Tc-and iron-based nanostructures.

### 3.3. Size-Dependent Effect of the CNCs on Spleen Cell Proliferation and Cytokine Production

**Figure 4:**
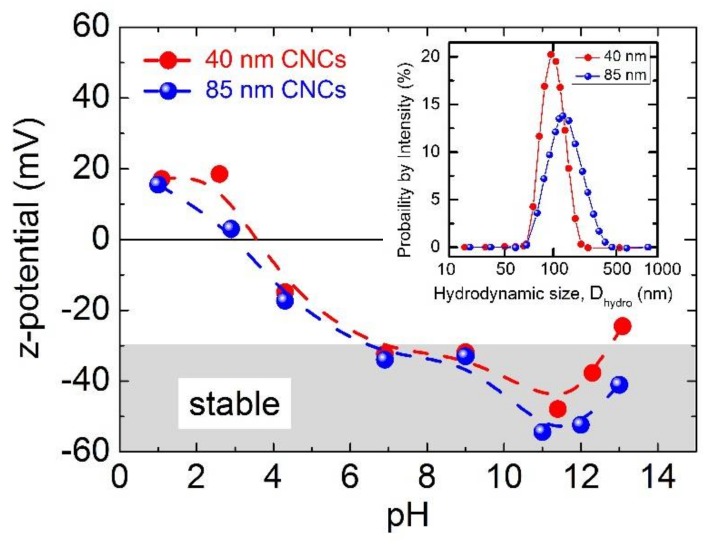
Effect of pH on the z-potential of 40-nm and 85-nm nanoclusters (CNCs). The shaded area indicates the region of colloidal stability. Inset: mean hydrodynamic diameters obtained by dynamic light scattering (DLS), weighted by intensity for the as-made purified samples, namely, D_hydro_ ≅ 93 nm (PdI ≅ 0.055) and D_hydro_ ≅ 125 nm (PdI ≅ 0.181), respectively (PdI = polydispersity index).

In order to characterize the nanocluster uptake at the cellular level and study possible size-dependent effects, we have chosen two nanoprobe specimens with D_TEM_= 40 ± 6-nm and D_TEM_= 85 ± 19-nm diameters (Figure S3). As the particle–cell interaction can be mediated by electrostatic effects as well, the two nanocluster samples were grown to attain similar surface charges (z-potential), namely of −45.1 mV and −45.9 mV, for the small (D_hydro_ ≅ 93 nm, at initial solution pH ≅ 7.2) and the large (D_hydro_ ≅ 125 nm, at initial solution pH ≅ 8.0) CNCs, respectively. For a first approximation then, any differing behavior in the bioreactivity of these iron oxide nanostructures may be assumed to arise from their size variation alone. Moreover, as the pH of the cellular environment has wide-ranging consequences in biological processes, and while the intracellular pH may be maintained around 6.5–7.5, there are cases where pH instabilities are likely. For this reason, an ex situ view of the stability of the nanoclusters under variable pH conditions becomes valuable. DLS experiments confirmed that aggregates (or agglomerates) of these nanoprobes are more likely to be formed at pH <4.5 and pH >12.5, while no dissolution in their individual nanocrystal subunits (or aggregation) is observed for any pH in between those two extremities (Figure S4). In fact, stable aqueous nanocluster colloids are anticipated in the pH scale of 5–12, as the z-potential is maintained in the desired region below about −30 mV (Figure 4).

**Figure 5:**
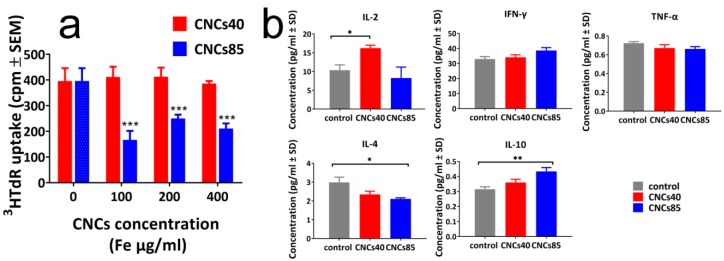
(**a**) Mouse spleen cell proliferation in the presence of CNCs of 40 nm and 85 nm in diameter was tested by ^3^HTdR incorporation assays 48 h after culture initiation. The results represent the mean of three different experiments and are expressed as cpm ± SEM; (**b**) Detection of cytokines in the supernatants of spleen cell cultures in the presence or not of CNCs at the concentration of 200 μg/mL. The results represent the mean of three experiments and are expressed as pg/mL ± SD. *: p < 0.01, **: p < 0.005, ***: p < 0.001 (see experimental).

With the evaluated pH stability of CNCs in hand, spleen cell proliferation was studied for three different nanocluster concentrations (100 μg Fe/mL, 200 μg Fe/mL, and 400 μg Fe/mL) for both samples (40-nm and 85-nm diameter) by evaluating the uptake of ^3^H-thymidine. The small nanoclusters did not affect the proliferation even for the highest concentration, while the larger ones showed a significant suppression of 57.8%, 36.9%, 46.7% for 100 μg Fe/mL, 200 μg Fe/mL, and 400 μg Fe/mL concentrations, respectively (Figure 5a). Furthermore, the nanoclusters of different sizes were found to exhibit a different behavior in the production of inflammatory and anti-inflammatory cytokines. The small nanoclusters were able to significantly increase the production of the inflammatory IL-2 (*p* = 0.0360), while the larger ones induced a significant decrease of anti-inflammatory cytokines IL-4 (*p* = 0.0250) and increase of IL-10 (*p*= 0.0023), arguing thus in favor of an immunostimulatory effect for the 40-nm nanoclusters, and an overall immunosuppressive effect for the 85-nm nanoclusters (Figure 5b). The production of IFN-γ and TNF-α was not altered by any of the cell treatments (Figure 5b).

**Figure 6:**
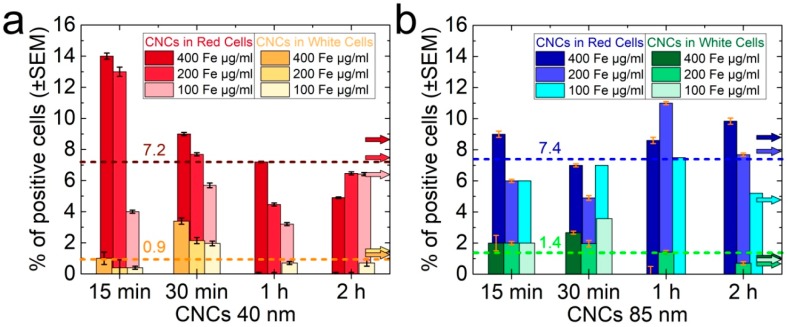
Uptake of CNCs [40 nm (**a**) and 85 nm (**b**)] by spleen cells incubated in their presence for 15 min, 30 min, 1 h, and 2 h, as well as for three different concentrations. The percent (%) averaged values have been indicated in the diagrams by the dashed lines. The percent (%) averaged values for each nanocluster concentration are also marked by the arrows.

Moreover, CNCs were tested for their ability to interact with red and white cells, which is an effect that could potentially lead to cell apoptosis or necrosis. Mouse spleen cells were incubated for 15 min, 30 min, 60 min, and 90 min with the nanoclusters at 37 °C in an atmosphere of 5% CO_2_. Nanocluster-bound cells were isolated by application of an external magnetic field to the cell-containing tube, washed, and subsequently counted. From this magnetically-driven procedure, only a small percentage of the total cell population (~5 to 15%) was shown to effectively bind nanoclusters. The average uptake for both red and white cells was marginally enhanced in the case of the larger nanoclusters (i.e., 7.4% and 1.4% versus 7.2% and 0.9% for the small ones) (Figure 6, dashed lines). Red cells preferentially absorbed the CNCs compared with white cells, which displayed their best absorbance during the first 30 min of incubation (Figure 6). Although small CNCs were absorbed by more red cells, these could rapidly lose their load (Figure 6a), while large CNCs, when absorbed by red cells, showed a more stable binding ability over time (Figure 6b). In contrast, only a small percentage of the white cells absorbed these iron oxide nanostructures. Their average uptake was 0.9% and 1.4% for the small and the large nanoclusters, respectively, showing a potentially increased interaction during the first 30 min of incubation; however, this rapidly decreased thereafter (Figure 6).

**Figure 7:**
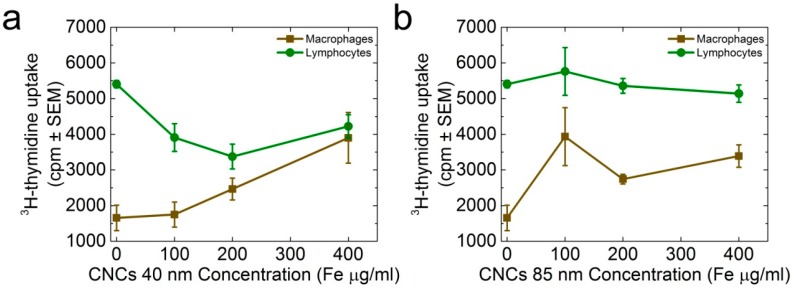
The proliferation of macrophages and lymphocytes incubated with CNCs [40 nm (**a**) and 85nm (**b**)] was evaluated against the uptake of ^3^H-thymidine (^3^HTdR).

Since white cells are active counterparts of an organism, it could be hypothesized that binding was undetectable after 30 min because of the internalization of such iron oxide nanoprobes into cells, thus necessitating a further exploration of this behavior. Consequently, macrophages and lymphocytes were isolated from spleen white cells, and were submitted to proliferation experiments in the presence (or not) of CNCs. As the likely ingestion mechanism of nanoparticles by cells is size-mediated [61], it is anticipated that both types of cells may receive CNCs by pinocytosis (<500 nm), while only macrophages can exert a phagocytic activity (>500 nm). Our experiments have shown that macrophages responded to CNCs of both sizes in a dose-dependent manner, and increased their proliferative ability as the nanocluster concentration was increased (Figure 7). On the other hand, lymphocytes decreased their proliferative activity in response to the 40-nm CNCs (Figure 7a), while they were insensitive in the presence of 85-nm CNCs (Figure 7b).

**Figure 8:**
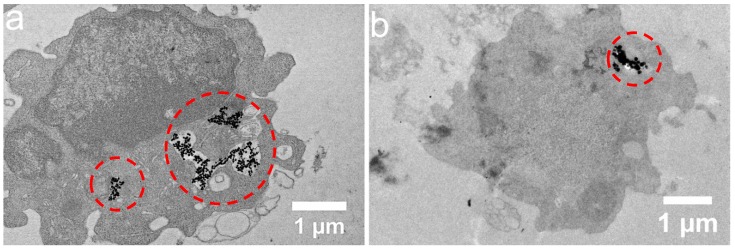
Bright-field TEM images of macrophages with 40 nm (**a**) and 85 nm (**b**) CNCs internalized (dashed circles) by likely endocytosis or engulfed inside endosomes.

In order to evaluate further the activity of macrophages and lymphocytes in response to the nanoclusters, cytokine production was also evaluated in the cultures of supernatants after 48 h of incubation. Although small CNCs were assumed to induce an immunostimulatory effect (produce IL-2 cytokines) and large CNCs caused an immunosuppressive effect in whole spleen cell cultures, when the major two populations of leukocytes were separated, macrophages were shown to increase IL-2 production in response to small and large nanoclusters (*p* = 0.0014 and *p* = 0.0040 respectively), while lymphocytes reduced IFN-γ production (*p* = 0.0097) only in response to small CNCs (Figure S5). Since immune responsiveness is subjected to cognitive recognition, the different cytokine profile in bulk spleen cultures and isolated cell populations is expected. Furthermore, as antigen-presenting cells, including macrophages, are the first cells to encounter the antigen and initiate an immune response, these were examined by transmission electron microscopy for their ability to ingest the CNCs. Indeed, TEM analysis showed that macrophages internalized both sizes of CNCs tested in this study; however, their morphological integrity is hard to be assessed from these low-resolution images (Figure 8a,b).

## 4. Discussion

The ferrimagnetic CNCs appear to satisfy the first prerequisite, namely that of magnetism engineered properties for improved MR detection capability. The capping of the nanoclusters with the polyacrylic acid is important, because it renders them negatively charged and highly dispersible/stable in their aqueous solutions, while it imparts them with an enhanced *r*_2_/*r*_1_ relaxivity ratio when they are compared against the former [46] commercial *T*_2_ contrast agent Endorem^©^ [45]. However, a second condition for their implementation in magnetically-driven applications requires the evaluation of the nanoclusters biodistribution, and the critical consideration of their interaction with cells. Importantly, nanomaterials of various compositions and diverse morphological and surface characteristics can interact with the immune system in several ways so that the immune function is either enhanced or suppressed.

Bearing in mind that it is crucial for CAs to be cleared from the body in a reasonable time frame [62], following their excretion via hepatic and renal (urine) elimination provides a means to assess the degree of their interaction (e.g., internalization/metabolism) with cells [63]. The continuous generation of new CAs for early diagnosis has shown that a promising pathway to reduce or slow down the severe and rapid reticuloendothelial system (RES) uptake is by tailoring the surface coating of inorganic nanoparticles [64]. Here, the high localization of the radiolabeled CNCs, in RES tissues such as the liver and spleen (Figure 2), appears as a limitation rather due to the fairly large size of the nanocarriers considered herein. The higher radioactivity in both organs, inferred a higher CNCs’ concentration, especially after the first hour of their intravenous injection in mice models. Radioactivity was observed neither in the thyroid gland nor in the stomach. The highest blood values were observed during the first 5 min, even though the percentage of CNCs was very low, indicating the fast elimination of CNCs from the blood circulation. This is consistent with previous findings, which suggest that nanosized inorganic particles above ~50 nm become distributed very fast in the liver and spleen [65], while smaller nanoprobes have larger blood half- lives, although they end up in the same RES organs [66]. The fast accumulation in the liver could infer that these nanomaterials may be of potential use in the diagnosis and therapy of liver and spleen diseases [67,68,69,70,71,72,73,74]. Another important experimental observation in view of the CNCs’ elimination from the organism is that such iron oxide nanostructures appear to reach the bladder in about 60 min (Figure 3), and are excreted via urine. Unless the overall particle size is reduced down to the renal-clearable scale (i.e., hydrodynamic diameter <6–8 nm) [75], where the filtration by the kidneys depends on the size/shape, charge, and surface ligand [63], switching from hepatic elimination to renal clearable nanocluster entities is unlikely. So, without being able to resolve the integrity of the CNCs’ morphological characteristics, but with iron-based species detected in the SEM-EDS of the urine specimen (Figure S2c,d), the possibility for renal excretion of the nanoclusters is likely to involve their dissociation into smaller fragments. Previous reports on ultrafine Au nanoparticles indicated a slower urinary excretion, reaching the bladder in 90 min [76]; in some cases, these were detectable in urine 30 days after their injection [77]. In another study, where iron oxide nanoparticles of 3 nm were utilized, a much slower excretion, apparently occurring mostly within the first 2.5 h, offers another demonstrated example of how elimination pathways are mediated by the particles’ chemically diverse surface coating [78].

An important question arising with the use of these technologies is whether CNCs may cause any cellular effects during their stay in the organism. Until excretion, these structures come in contact with various cell types in the blood and tissues. Therefore, the present work examined whether CNCs can be taken up by immune system cells and in turn regulate or alter their function. The choice of immune cells was based on their ability to direct an accurate and rapid response to external stimuli, which in turn may initiate a cascade of events in favor of or against the host. To this extent, total spleen cells were shown to decrease proliferation in response to small, but not large, CNCs. Small CNCs are most probably ingested by the cells in a clathrin and caveolin-independent manner, and therefore can easily affect cell functions. Indeed, it was shown that 40-nm CNCs could significantly increase IL-2 production, probably towards immune activation. On the other hand, 85-nm CNCs, although not affecting cell proliferation, decreased IL-4 and increased IL-10 production in bulk spleen cell cultures, driving the immune system toward an immunosuppressive state. This result is a size-dependent feature, and is thus not due to the different surface environment of the nanoprobes. It is worth noting here that the surface charges for both specimens of CNCs are identical. Also, in other studies of iron oxide nanoparticles, with diameters of 10-nm and the same capping agent, inflammatory cytokine activation was also detected [79].

It is interesting to note that a higher percent of red cells could interact with CNCs compared with white cells. However, this is most likely because the red cells are rather inactive, and are unable to proceed with the internalization/metabolism of the CNCs, and as such, the inorganic nanostructures could by detected to reside at the cell membrane for a longer period of time. On the contrary, it can be hypothesized that the white cells rapidly ingested CNCs, and therefore membrane interaction lasted only for a short period of time.

In an attempt to separately study the immune cell populations, the macrophages and lymphocytes of white cells were independently treated with CNCs. In this case, a differential activity was detected amongst the separated cell types. The proliferative ability of macrophages increased in the presence of both sizes of CNCs, which was also accompanied by increased levels of IL-2 production. In contrast, lymphocytes showed decreased proliferation only in response to the 40-nm CNCs, which was accompanied by the decreased production of IFN-γ. Although these results are interesting, it is obvious that cellular behavior in isolated cell populations is different from that in bulk cell cultures, where cell–cell interactions are allowed. CNCs were indeed internalized by macrophages, as demonstrated by TEM analysis, which would probably emit a signal-altering lymphocyte responsiveness.

Following the course of dispersion of these iron oxide nanoprobes within the organism, the present study demonstrates that liver and spleen were the primary sites of the nanocluster accumulation. However, their passage through the different organs is apparently not indifferent to cellular responses. The close uptake of CNCs from immune cells may be manipulated in favor to the host. Thus, small-sized CNCs seemed to drive immunostimulatory responses, while larger CNCs could lead to immunosuppressive behavior. Such observations give new insight in iron oxide (maghemite) nanocluster applications for size-controlled regulation of the immune system.

## 5. Conclusions

In summary, facile chemical concepts developed for the controlled clustering of iron oxide interacting nanocrystals afford tailored nanoprobes with unique magnetic features. These nanoassemblies, despite their large volume, present biological utilities beyond those set out by their bare magnetism-engineered nature. With their size controlled and the surface functional groups tailored, the pomegranate-like morphology maghemite nanoclusters (40–85 nm) discussed here can be utilized as a multifunctional platform that facilitates magnetically-driven applications, such as magnetic resonance imaging (MRI), and the potential localized treatment of tumor matrixes via magnetic hyperthermia. Careful monitoring of the distribution, circulation, and likely excretion of such nanoscale vectors through the organism helps to infer effectiveness towards their preclinical use as contrast agents for diagnosis of liver lesions and for cell-based therapy (immunotherapy or stem cell therapy) in related diseases. However, the endeavor to elucidate mechanisms in vivo through non-invasive MRI and nuclear imaging modalities requires improving practical treatment plans to adapt to the complexity of the targeted physiological environment. Although these nanoprobes attain relatively similar MRI capabilities, nanoclusters of different sizes appear to present a crucially unlike degree or type of interaction at the cellular level. Without comprising the power of therapeutic drug molecules, smaller size nanoclusters might be effective for therapeutic purposes where the activation of the immune system is required, whereas larger nanoplatforms appear potentially promising as immune-suppressive agents. The ability of the nanoclusters to induce size-dependent cytokine production, although it may alleviate immune-related theranostics, necessitates intense preclinical efforts to uncover cellular processing mechanisms under which the nanoclusters’ bioactive modalities become a tool that battles diseases in a targeted fashion.

## Supplementary Materials

The following are available online at http://www.mdpi.com/2079-4991/8/5/315/s1. Figure S1: Stability study of the radiolabeled CNCs; Figure S2: Optical microscopy and SEM-EDS analysis of a specimen after renal (urine) excretion of the studied iron-oxide nanostructures; Figure S3: Nanoclusters’ TEM images and size distributions; Figure S4: pH variation of the CNCs size distribution as determined by DLS; Figure S5: Cytokine generation in lymphocytes and macrophages isolated from spleen white cells.
