# Supplementary material for: Iron Oxide Colloidal Nanoclusters as Theranostic Vehicles and Their Interactions at the Cellular Level
Source: Nanomaterials (Basel). 2018 May 9;8(5):315. doi: 10.3390/nano8050315 (PMC5977329; doi:10.3390/nano8050315)
Supplement: Supplementary file 1 [file nanomaterials-08-00315-s001.docx]

-Supporting Information-

Iron-oxide Colloidal Nanocluster as Theranostic Vehicles and their Interactions at the Cellular Level

Athanasia Kostopoulou^1^, Konstantinos Brintakis^1^, Eirini Fragogeorgi^2^, Amalia Anthousi^3^, Liberato Manna^4^, Sylvie Begin-Colin^5^, Claire Billotey^6^, Anthi Ranella^1^, George Loudos ^7,8^, Irene Athanassakis^3^ and Alexandros Lappas^1,^*

^1^ Institute of Electronic Structure and Laser, Foundation for the Research and Technology – Hellas, Vassilika Vouton, 711 10 Heraklion, Greece; akosto@iesl.forth.gr (A.K.); kbrin@iesl.forth.gr (K.B.); ranthi@iesl.forth.gr (A.R.)

^2^ Institute of Nuclear & Radiological Sciences, Technology, Energy & Safety, NCSR “Demokritos”, 153 41 Aghia Paraskevi – Athens, Greece; fragkogeorgi@rrp.demokritos.gr

^3^ Department of Biology, University of Crete, Vassilika Vouton, 710 03 Heraklion, Greece; Amalia.Anthousi@lstmed.ac.uk (A.A); athan@biology.uoc.gr (I.A.)

^4^ Istituto Italiano di Tecnologia, Via Morego 30, 16163 Genova, Italy; liberato.manna@iit.it

^5^ Université de Strasbourg, CNRS, Institut de Physique et Chimie des Matériaux de Strasbourg, UMR 7504, F-67034 Strasbourg, France; sylvie.begin@ipcms.unistra.fr

^6^ Université de Lyon – Université Jean Monnet, EA 3738 – Ciblage Thérapeutique en Oncologie – UJM-UCBL-HCL, Hôpital E. Herriot, 5 place d’Arsonval – 69437 Lyon CEDEX 03, France; claire.billotey@univ-st-etienne.fr

^7^ Bioemission Technology Solutions, Alexandras 116, 117 42 Athens, Greece; gloudos@teiath.gr

^8^ Department of Biomedical Engineering, Technological Educational Institute, 122 10 Egaleo – Athens, Greece

***** Correspondence: lappas@iesl.forth.gr; Tel.: +30-2810-39-1344


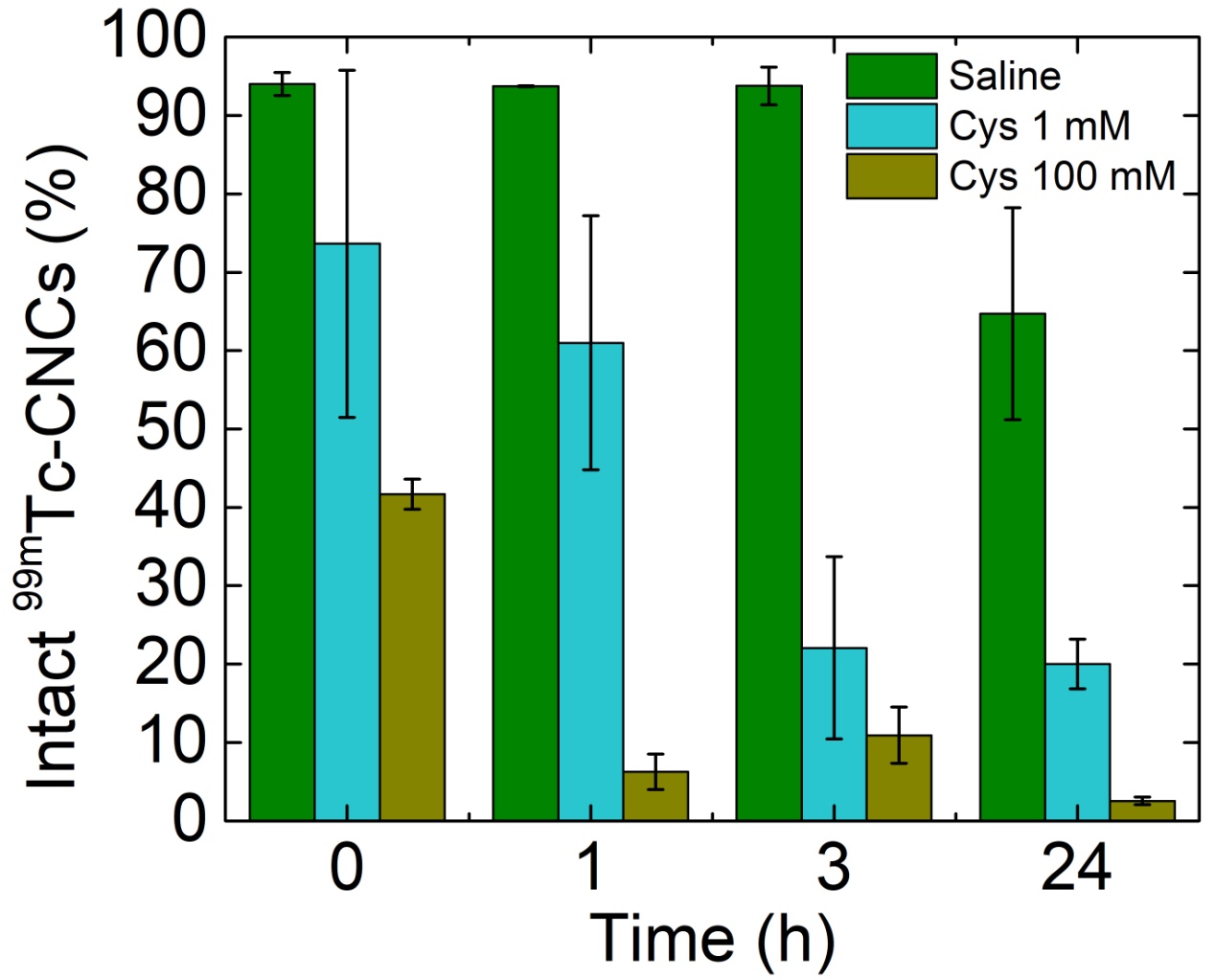


**Figure S1.** Percentage of intact radiolabeled nanoclusters (D_TEM_~ 73 nm) at 1, 3 and 24 h post-preparation (p.p.) under aqueous solution (NaCl 0.9% v/v)) and against trans-chelation (1 mM and 100 mM Cysteine solution); (means ±standard deviation, (n=2)).


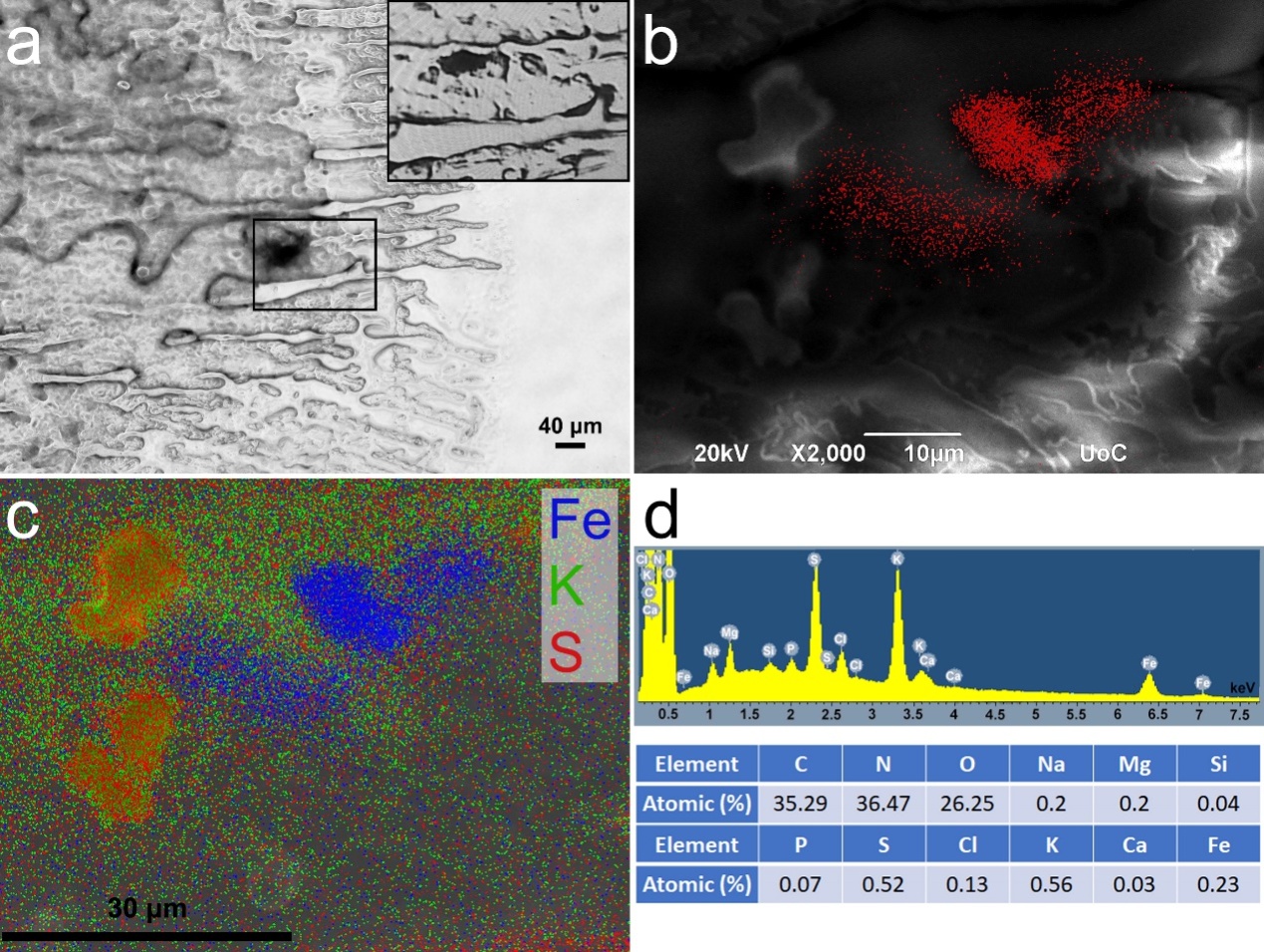


**Figure S2.** Images from an optical microscope (a), a scanning electron microscope with the superimposed EDS mapping (only for the Fe content – regions colored in red ) (b) of the dried urine collected after 1 h from the intravenous injection of the CNCs in a mouse model. Elemental mapping and EDS analysis obtained from the same area (c, d).


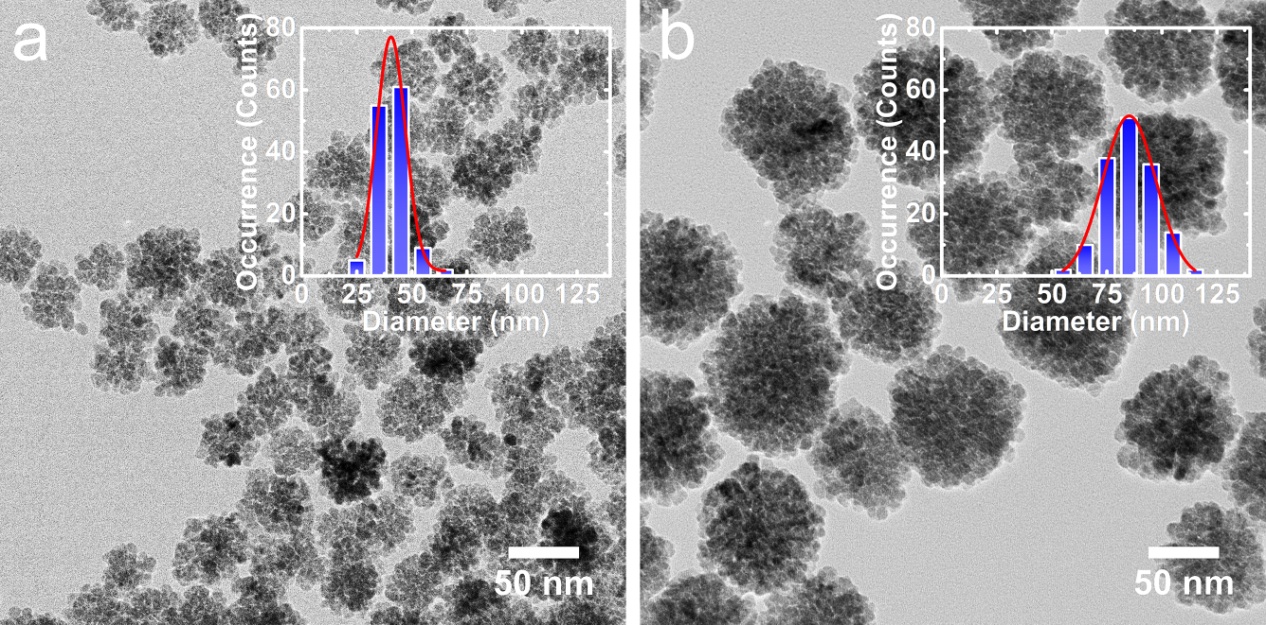


**Figure S3.** Low magnification TEM images and size distribution of specimens entailing 40 (a) and 85 (b) nm diameter nanoclusters.


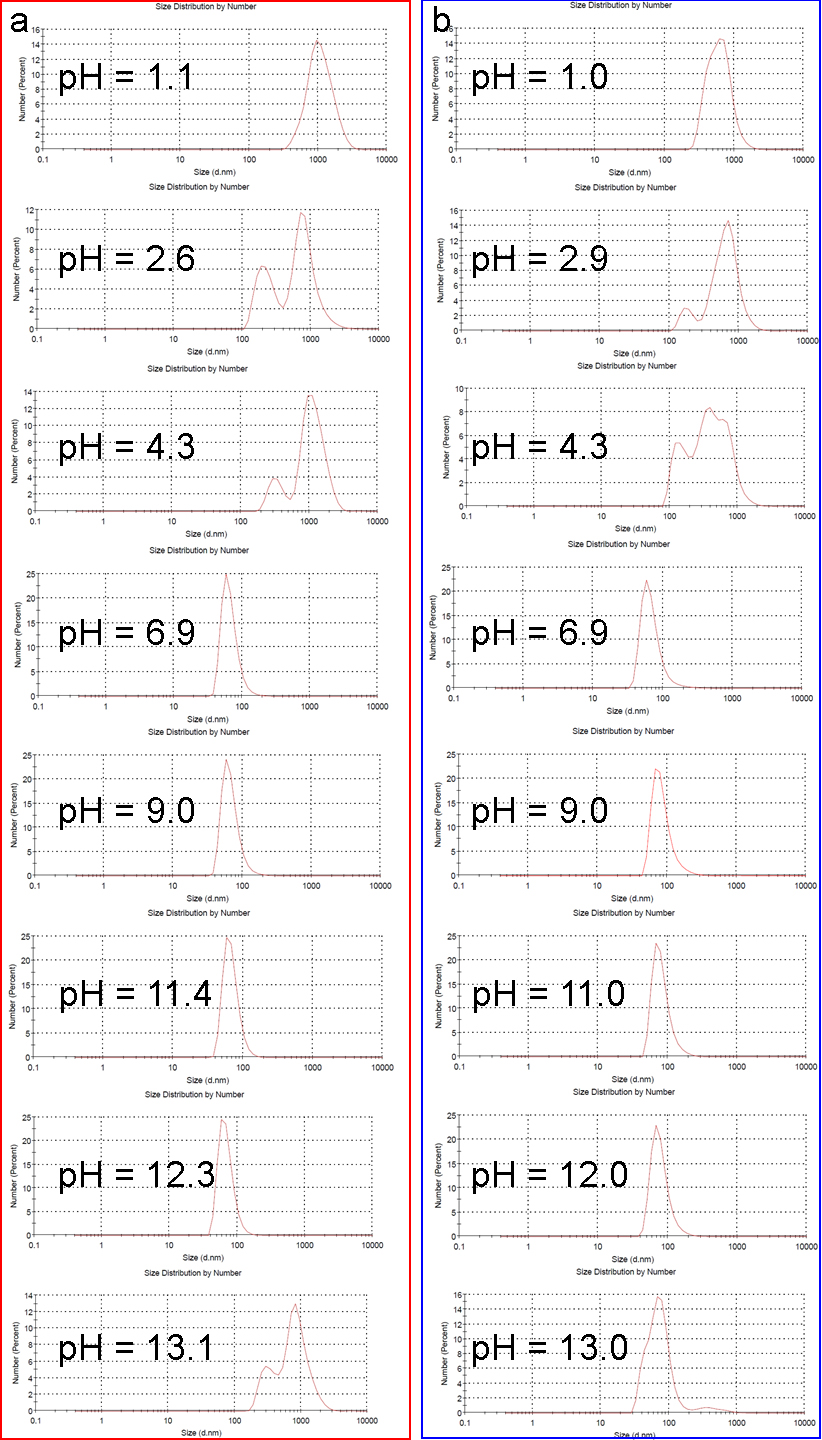


**Figure S4.** The size distribution of the 40 nm (a) and 85 (b) nm nanoclusters as a function of the colloidal solution pH variation, characterized by Dynamic Light Scattering (DLS) weighted by number.

**
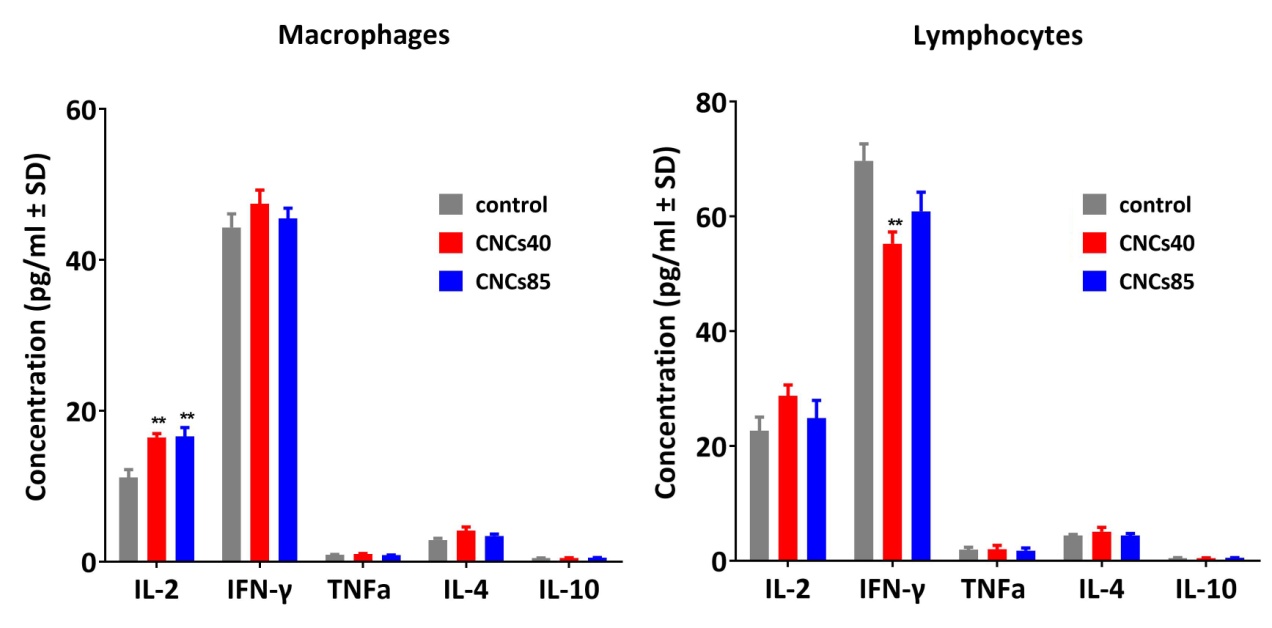
**

**Figure S5.** Cytokine content in lymphocytes and macrophages isolated from spleen white cells. The latter were cultures of supernatants incubated for 48 hr in the presence of 40 and 85 nm CNCs (200 Fe μg/ml). The results represent the mean of 3 experiments and are expressed as pg/ml ± SD. ***: p*<0.005.
